# ConGEMs: Condensed Gene Co-Expression Module Discovery Through Rule-Based Clustering and Its Application to Carcinogenesis

**DOI:** 10.3390/genes9010007

**Published:** 2017-12-28

**Authors:** Saurav Mallik, Zhongming Zhao

**Affiliations:** 1 Department of Computer Science & Engineering, Aliah University, Newtown, WB-700156, India; sauravmallikr2@gmail.com; 2 Center for Precision Health, School of Biomedical Informatics, The University of Texas Health Science Center at Houston, Houston, TX 77030, USA; 3 Human Genetics Center, School of Public Health, The University of Texas Health Science Center at Houston, Houston, TX 77030, USA

**Keywords:** gene co-expression modules, Limma, association rule mining, dynamic tree cut method, gene expression markers, lung squamous cell carcinoma

## Abstract

For transcriptomic analysis, there are numerous microarray-based genomic data, especially those generated for cancer research. The typical analysis measures the difference between a cancer sample-group and a matched control group for each transcript or gene. Association rule mining is used to discover interesting item sets through rule-based methodology. Thus, it has advantages to find causal effect relationships between the transcripts. In this work, we introduce two new rule-based similarity measures—weighted rank-based Jaccard and Cosine measures—and then propose a novel computational framework to detect condensed gene co-expression modules (ConGEMs) through the association rule-based learning system and the weighted similarity scores. In practice, the list of evolved condensed markers that consists of both singular and complex markers in nature depends on the corresponding condensed gene sets in either antecedent or consequent of the rules of the resultant modules. In our evaluation, these markers could be supported by literature evidence, KEGG (Kyoto Encyclopedia of Genes and Genomes) pathway and Gene Ontology annotations. Specifically, we preliminarily identified differentially expressed genes using an empirical Bayes test. A recently developed algorithm—RANWAR—was then utilized to determine the association rules from these genes. Based on that, we computed the integrated similarity scores of these rule-based similarity measures between each rule-pair, and the resultant scores were used for clustering to identify the co-expressed rule-modules. We applied our method to a gene expression dataset for lung squamous cell carcinoma and a genome methylation dataset for uterine cervical carcinogenesis. Our proposed module discovery method produced better results than the traditional gene-module discovery measures. In summary, our proposed rule-based method is useful for exploring biomarker modules from transcriptomic data.

## 1. Introduction

Whole transcriptome data has been growing rapidly over the past two decades. Such data have been used extensively for detecting the altered expression of genes, leading to one of a set of gene signatures for complex diseases such as cancer, diabetes, and neurodevelopmental disease [1,2,3,4,5,6,7]. Genome-wide analysis of differential mRNA expression has been useful in identifying informative genes, biological pathways, and molecular networks involved in diseases. To comprehend the complexity of the transcriptomic study, microarray labeling data [1,8] are useful. Microarray labeling essentially measures the difference between a group of cancer samples and a group of control (non-cancerous) samples for each transcript (gene). Association rules are used to analyze the microarray labeling data. Association rules are composed of if/then statements that describe the relationship between an antecedent (if) found in the data and a consequent (then) associated with it. A rule is associated with various rule-interestingness measures. Among these, support (frequency) and confidence (strength) are general rule-interestingness measures. The support of an item set is equal to the number of transactions in which all items belonging to the item set occur simultaneously. An item set becomes frequent if its support is greater than or equal to any user-specified cutoff value (denoted as minimum support). The confidence of a rule is the ratio of support of the whole item set to the support of its left hand side (i.e., antecedent). Strong rules exceed a minimum threshold for support and confidence [9,10].

Association rule mining [9,10,11] is a well-known technique for exploring interesting relationships, associations, or cause–effect structures between various items (genes) in different types of databases (viz., transactional databases, relational databases, and other types of data repositories). For example, based upon the idea of strong rules, Agrawal et al. [9] proposed association rules for identifying the associations between the sale products (items) in the large transactional database documented through the point-of-sale (POS) systems in supermarkets. For example, a rule {bread,butter}⇒{sugar} found in the record of sale data repository of the supermarket indicates that if a customer purchases bread and butter simultaneously, it is likely that the customer will also purchase sugar. This type of information is used to make decisions regarding placement of products as well as promotional pricing. Association rules are also utilized in various application domains including bioinformatics, web-based mining, intrusion detection, etc. [1,11]. To generalize, let $IT=\{i_{1},i_{2},\ldots ,i_{n}\}$ be an item set (gene set) and $T=\{t_{1},t_{2},\ldots ,t_{m}\}$ be a set of transactions (samples). Therefore, a rule can be defined such that A⇒C. Here A,C⊆IT and A⋂C=ϕ. *A* stands for antecedent (left hand side or LHS), whereas *C* refers to consequent (right hand side or RHS). This rule states that if all items belonging to *A* occur together, it is likely that all the items belonging to *B* also occur simultaneously. Similarly, in the case of the gene expression [12] dataset, a set of genes might occur simultaneously in a sample. Among them, some might be up-regulated, some down-regulated, whereas the rest are non-differentially expressed. For example, let $\left\{g1+,g2-,g3_{nonde}\Rightarrow g4-\right\}$ be an association rule that states that if gene *g*1 is up-regulated (represented by “+”), gene *g*2 is down-regulated (represented by “−”), and gene $g3_{nonde}$ is non-differentially expressed (“nonde”) in the same sample, it is likely that gene *g*4 will be down-regulated.

In network biology, a gene co-expression module refers to a group of genes whose expression is highly related to the phenotype being studied and whose co-expression is highly related or significant [13,14,15,16]. According to literature, opinions differ on the definition of a gene-module (e.g., [17,18,19,20]). Co-expression is a term that is highly useful in gene-module recognition. Co-expression between genes states that these gene expression profiles might concurrently rise and fall during a sub-span of time-series rather than the complete time-series.

Cancer is one of the most prevalent human diseases, and its underlying biology is complex. Lung squamous cell carcinoma (LUSC) is a cancer subtype found in about 40% of lung cancer patients. LUSC patients are often diagnosed at a late stage. Various gene-markers have been identified for LUSC using different genomic and genetic datasets, including somatic mutations (single nucleotide variants, copy number variations, gene expression, methylation, gene fusion, etc.) and germline mutations (e.g., genome-wide association studies) [21]. Many approaches and tools have been used for genetic marker discovery; however, genetic-rule-module approach—which can improve the correctness and efficiency of the interactive mechanisms of the genes in the disease phenotypes—has not yet been applied. In this manuscript, we presented a new computational framework for detecting condensed gene co-expression modules (ConGEMs) through association rule-based learning. In this framework, we first applied an empirical Bayes test on the normalized gene expression data through Limma software [22] for obtaining differentially expressed genes (DEGs) [1,23] across two types of samples. The DEGs were then discretized and post-discretized consecutively to convert them into the corresponding boolean forms. Thereafter, we applied a recent weighted rule mining algorithm, RANWAR [10], for generating the association rules. We then introduced two novel weighted rank-based similarity measures (viz., weighted rank-based Jaccard and Cosine measures) between two rules. Thereafter, we computed the integrated similarity score of these newly introduced weighted rule-based similarity measures among the pairwise rules, and then applied the average linkage clustering using the integrated score. Dynamic tree cut method [24,25] was then utilized on the resultant dendrogram of the clustering for recognizing co-expressed rule-modules. In addition, we identified the list of evolved condensed markers that consists of both singular and complex markers in nature depending upon corresponding condensed genesets in either antecedent or consequent of the rules of the resultant modules. We next evaluated those genes through literature search, KEGG pathway and Gene Ontology (GO) analyses. Depending upon these, we assessed our method by both known markers and novel markers. We applied our framework to a gene expression dataset for LUSC as well as a methylation dataset for uterine cervical carcinogenesis. Finally, we provided a comparative study between the rule-module identification in our proposed method and the other traditional gene-module identification measures in terms of several cluster-validity measures. Moreover, our proposed method is useful for  similar datasets in the study of other respective diseases.

## 2. Materials and Methods

### 2.1. Literature Review

Transcriptome study is able to detect genes which are differentially expressed in specific tissues, and also assists in finding potential disease markers. Microarray labeling data is frequently used for analyzing the transcriptome. Here, DEGs can be identified using different statistical testing methods, such as *t*-test, empirical Bayes test, SAM (significance analysis of microarrays), among others [26].

Detecting genetic markers from genomic data like microarray gene expression has long been useful in biomedical research [27]. To date, most studies have focused on single gene analysis [1]. The gene co-expression network of identified genes will represent the blueprint of the inter-connections between them, along with the molecular regulators (i.e., transcription factors (TFs) and microRNAs) [28]. A gene co-expression module in network biology refers to a group of genes whose expression is highly related to the phenotype under examination and whose co-expression is highly related or significant [13,28]. From the literature, many measures regarding gene-module detection have already been proposed [29]. The topological overlap measure (TOM) [29] is one such measure. The TOM score between two nodes (*i* and *j*), symbolized as TOM(i,j), is defined as:

(1) $$TOM\left(i,j\right)=\left\{\begin{array}{cc} \frac{\sum_{u\neq i,j}X\left(i,u\right)X\left(j,u\right)+X\left(i,j\right)}{min\{\sum_{u\neq i}X\left(i,u\right),\sum_{u\neq j}X\left(j,u\right)\}-X\left(i,j\right)+1}, & \mathrm{if}\;i\neq j,\\ 1, & \mathrm{if}\;i=j, \end{array}\right.$$

where *X* stands for the corresponding adjacency matrix having binary entries, “1” and “0”. An entry of “1” states that the two nodes have a direct connection, whereas an entry of “0” signifies that there is no direct connection between these two nodes.

In addition, in the literature, several updated versions of TOM such as weighted TOM (wTOM) [25,30], generalized TOM of degree “*m*” (GTOM0) [31] (where “*m*” denotes 0, 1, 2, ...) exist. To compute the wTOM, the Pearson’s correlation coefficient (PCC) or Spearman’s correlation (SC) is first calculated for all node-pairs. Thereafter, the power by which the adjacency matrix will be computed from the correlation matrix is determined by soft thresholding [30]. The adjacency matrix is then computed from the adjacency matrix using that power. After that, wTOM scores are evaluated. After obtaining the wTOM/GTOMm matrix, the distance matrix is then computed. Average linkage clustering and dynamic tree cut are then applied respectively. As a result, gene-modules denoted by different colors are produced.

While there are many biomarker studies (including some that are gene-module based), little work has been done based on rule-module detection. Here, a rule comprises several genes. Sethi et al. (2010) [32] attempted to propose weighted rule-based similarity measures, but they did not apply their rule-module detection to specific biomarker discovery. Hence, in this manuscript, we adapted the measures by Sethi et al., and developed a new computational framework—ConGEM—for detecting condensed co-expressed rule-modules through the modified weighted similarity measures. Thereafter, we identified the list of evolved condensed markers that consists of both singular and complex markers in nature depending upon corresponding condensed gene sets in either the antecedent or consequent of the rules of the resultant modules. Furthermore, we identified the list of evolved condensed markers consisting of both singular and complex markers depending upon corresponding condensed genesets in either antecedent or consequent of the rules of the resultant modules. Moreover, we evaluated the resultant rule-modules through the several standard cluster validity indices such as Dunn index, Silhouette width, scaled connectivity, clustering co-efficient, etc. Additionally, we included the comparative study between the resultant rule-modules in our proposed method and the traditional aforementioned gene-module detection measures such as wTOM, GTOMm (where *m* = 0, 1, 2, 3).

### 2.2. Proposed Method of Identifying Condensed Rule-Modules

In this manuscript, we propose a new approach for identifying condensed gene co-expression rule-modules through rule-based learning. In this regard, we introduced two new similarity measures (viz., weighted support and weighted cosine) described in Section 2.2.6 inspired by Sethi et al.  [32], and then we applied these measures in our proposed rule-module detection. Moreover, all the major steps of the proposed methodology are provided briefly in Algorithm 1 for clear visualization. The steps of the proposed method were:
**Algorithm 1**
ConGEM  **Inputs:** (pvalue_threshold, min_wsupp, min_wconf, number_ERules), where pvalue_threshold refers to the user-specified cutoff for the corrected *p*-value, min_wsupp denotes the user-defined cutoff for (weighted) support value (i.e., minimum support), min_wconf stands for the user-defined cutoff for (weighted) confidence value (i.e., minimum confidence), and number_ERules be the user-notified number of top (experimental) rules on which the similarity matrix will be computed.  **Outputs:** (Co-expressed rule-modules, Cluster-validity indices, Condensed markers).
1:Normalize the input raw data-matrix gene-wise using zero-mean normalization.2:Utilize Limma to perform non-parametric empirical Bayes test and correct *p*-values using Benjamini–Hochberg fdr (false discovery rate)  method consecutively on the normalized data in order to identify differentially expressed genes. Let the resultant matrix having only the statistically significant genes be symbolized as *I*.3:Rank the resultant genes (i.e., $rank_{i}$) with respect to their corrected *p*-value in the empirical Bayes test.4:Assign weight (i.e., $w_{i}$) to each gene based on its rank $rank_{i}$.5:Transpose the normalized matrix *I*.6:Discretize the transposed matrix $I^{\prime }$ and identify resultant matrix DI in such a way that if $I^{\prime }\left(i,k\right)>=0$, then DI(i,k)←1, else DI(i,k)←0.7:Double the number of columns (genes) of DI, and then carry out post-discretization step in such a way that if DI(i,k)==1, then PDI(i,k)←1; and if DI(i,k)==0, then PDI(i,k)←0 for the first half; and thereafter for the second half, DI(i,k)==1, then $PDI(i,re_{n}+k)\leftarrow 0$; and if DI(i,k)==0, then $PDI(i,re_{n}+k)\leftarrow 1$, where $re_{n}$ is the number of *p*-value significant genes in the test.8:Apply RANWAR rule mining algorithm for identifying association rules with user-defined min_wsupp and min_wconf.9:Compute the proposed weighted Jaccard (i.e., Wζ) and weighted Cosine (i.e., WCos) similarity values among the pairwise rules of L1.10:Integrate Wζ and WCos values, and obtain integrated similarity values (i.e., Int_WζCos) for each rule-pair (e.g., $r_{1}$ and $r_{2}$). Find dissim values from these Int_WζCos values.11:Perform average linkage clustering method using dissim values for each rule-pair, and obtain dendrogram of the clustering.12:Utilize dynamic tree cut method on the resultant dendrogram, and identify the condensed gene co-expressed rule-modules (ConGEMs).13:Additionally, determine the frequency of the geneset of each side of every rule in same module, and identify the most frequent geneset from antecedent as well as the most frequent geneset from consequent for each rule-module.14:List all the resultant genesets, and then identify the top potential condensed gene expression genesets as top condensed gene expression markers for the disease.The condensed marker is based on “geneset equivalent pruning regulations” that are mentioned in the following: (i) *Geneset Equivalent Pruning regulation 1:* If there are some genesets whose genes are overlapped with each other (e.g., “a” and “a, b”), and if each of the genesets have the same frequency, then we consider only the geneset (e.g., “a, b”) that covers all the participating genes belonging to all these genesets, and subsequently the remaining genesets (e.g., “a”) are eliminated from the list. (ii) *Geneset Equivalent Pruning regulation 2:* If there are some genesets whose genes are overlapped with each other (e.g., “a” and “a, b”) and if each of the genesets contain different frequency, then we consider only the geneset among them which has highest frequency, and subsequently remaining genesets are omitted.15:Perform literature search as well as KEGG pathway and Gene Ontology analyses for the verification of identified Condensed Markers (CGMs).16:Evaluate the rule-module through several standard cluster validity indices such as Dunn index, clustering co-efficient, silhouette width, scaled connectivity, etc.


#### 2.2.1. Identification of Differentially Expressed Genes

First, some genes having low variance were eliminated from the gene expression data. Thereafter, we performed zero-mean normalization for the data of these genes so that the scale of the values across the datasets will be the same. The zero-mean normalization is defined as follows:

(2) $$x\_norm\left(i,k\right)=\frac{x(i,k)-\mu }{\sigma },$$

where x(i,k) denotes the value of the *i*-th gene at the *k*-th sample before normalization, x_norm(i,k) denotes the value of the *i*-th gene at the *k*-th sample after normalization, μ and σ depict mean and standard deviation, respectively, of the data of the *i*-th gene before normalization.

To identify differentially expressed genes (denoted by DEGs) among samples, an appropriate statistical test was needed. Since the Limma package—based on empirical Bayes test [22]—performs well for all sizes of data (i.e., data with small/medium or large sized samples) and any type of data distribution (normal/non-normal distribution), we have applied it here. The genes were thereafter ranked by their *p*-values. See details about Limma and the identification of the differentially expressed genes in Supplementary File S13.

After obtaining the *p*-value of the genes, we further computed a Benjamini–Hochberg corrected *p*-value (i.e., false discovery rate, FDR) to address the multiple test issues. Now, the genes whose *p*-values were less than 0.05 were called as DEGs, whereas remaining genes were simply insignificant genes, and were thus removed from the experimental analysis. The resultant corrected *p*-value significant genes were then ranked with respect to their corrected *p*-value in the test (e.g., $rank_{i}$, where 1≤i≤re_n, and re_n refers to number of *p*-value significant genes in the test). Let, *I* be the data matrix of the resulting genes having size re_n×m, where *m* is number of samples.

#### 2.2.2. Assigning Gene-Based Weight

In any genome-wide biological data, the importance of all genes in their roles varies substantially. A weight was assigned to each gene according to its corrected *p*-value ranking in the Limma test. The weight of each gene (i.e., $w_{i}$) was computed by a function of $rank_{i}$ and the lowest rank among the resultant DEGs (denoted by rankmax), as described below:

(3) $$w_{i}=\frac{1}{rankmax}*\left(rankmax-\left(rank_{i}-1\right)\right).$$

#### 2.2.3. Discretization

Since $I^{\prime }$ (i.e., transposed *I* matrix) was already normalized using zero-mean normalization, the remaining step for discretization of $I^{\prime }$ was

(4) $$DI\left(i,k\right)=\left\{\begin{array}{cc} 1, & \mathrm{if}\;I^{\prime }\left(i,k\right)>0,\\ 0, & \mathrm{if}\;I^{\prime }\left(i,k\right)<0, \end{array}\right.$$

where DI is the discretized matrix. Let $DEG_{up}$ and $DEG_{down}$ denote the up-regulated and down-regulated genes, respectively. Equation (4) demonstrates that any cell-value of $I^{\prime }$ that is greater than the mean value 0 is reassigned to 1 (referred to as $DEG_{up}$ gene), otherwise 0, (referred to as $DEG_{down}$ gene).

#### 2.2.4. Post-Discretization

**Figure 1:**
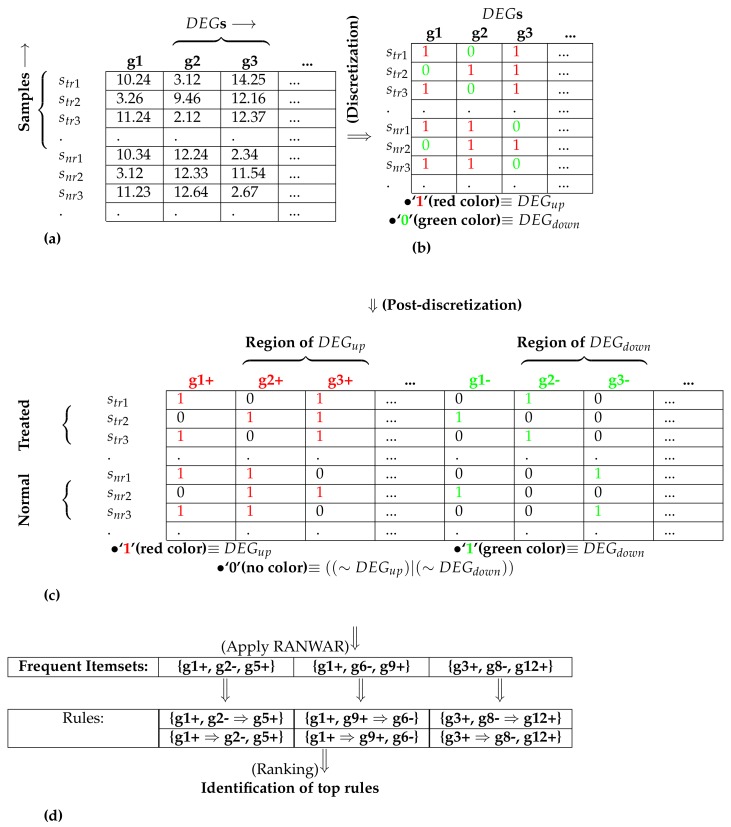
An example for performing the post-discretization: sub-figure (**a**) denotes the initial matrix containing differentially expressed genes (DEGs); (**b**) represents the matrix after discretization; (**c**) depicts the matrix after post-discretization; (**d**) signifies the utilization of association rule mining and the identification of top rules, where “+” refers to up-regulation (also denoted by “1”), and “−” refers to down-regulation (also denoted by “0”), $s_{tr}$ denotes diseased/treated samples, and $s_{nr}$ denotes normal samples.

We now have four categories of genes: (i) $DEG_{up}$ (denoted by 1 in DI); (ii) $DEG_{down}$ (denoted by 0 in DI); (iii) $∼DEG_{up}$; and (iv) ∼$DEG_{down}$. As any rule mining technique considers only 1, not 0, post-discretization was required in order to represent the aforementioned four types of genes using only two binary digits (i.e., 1 and 0). For the post-discretization, the number of columns (genes) was doubled while the first half of them denoted the region of $DEG_{up}$, and the second half of them denoted the region of $DEG_{down}$. For the first half, 1 showed $DEG_{up}$ property, and 0 denoted ∼$DEG_{up}$ property (see Equation (5)). For the second half, 1 showed $DEG_{down}$ property, and 0 denoted ∼$DEG_{down}$ property (see Equation (6)).

(5) $$PDI\left(i,k\right)=\left\{\begin{array}{cc} 1, & \mathrm{if}\;DI(i,k)=1,\\ 0, & \mathrm{if}\;DI(i,k)=0, \end{array}\right.$$

(6) $$PDI\left(i,re\_n+k\right)=\left\{\begin{array}{cc} 0, & \mathrm{if}\;DI(i,k)=1,\\ 1, & \mathrm{if}\;DI(i,k)=0, \end{array}\right.$$

where PDI shows the m×(2∗re_n) post-discretized matrix (see Figure 1). Here, “+” and “−” refer to up-regulation and down-regulation of any gene, respectively.

#### 2.2.5. Identification of Weighted Association Rules

After post-discretization, we identified the weighted association rules using a recently published rule mining technique called RANWAR [10]. The details of RANWAR are depicted in Supplementary File S16. In brief, user-defined support threshold and confidence threshold (denoted by min_wsupp and min_wconf) were provided to the RANWAR tool, and the gene-association rules identified. As an example of {g1+,g2−⇒g5+}, if gene 1 was upregulated and gene 2 was downregulated simultaneously in the same data, gene 5 would be upregulated.

#### 2.2.6. Proposed Weighted Rule-Based Similarity Measures

For the purpose of grouping the evolved rules in terms of their similarity values, we proposed two weighted rule-similarity measures, weighted Jaccard and weighted Cosine, to compute the genes’ correlations on the basis of similarity of two rules (vectors) (e.g., $r_{1}$, $r_{2}$). The two rules were as follows: $\left\{r_{1}^{a}\to r_{1}^{c}\right\}$ and $\left\{r_{2}^{a}\to r_{2}^{c}\right\}$.

**Definition** **1.** *The weighted Jaccard measure (i.e., $W\zeta (r_{1},r_{2})$) between the two rules is defined as the ratio of the weighted sum of common genes either on the same side of both of the rules (i.e., left hand side (LHS) or right hand side (RHS) of both the rules) or on the opposite side (i.e., LHS of one rule and RHS of the other rule) to the weighted sum of the union of genes existing either on the same side of both rules or on the opposite sides (Equation (7)).*

(7) $$W\zeta \left(r_{1},r_{2}\right)=\frac{\left(\sum_{p=1}^{|r_{1}^{a}\cap r_{2}^{a}|}w_{p}+\sum_{p=1}^{|r_{1}^{c}\cap r_{2}^{c}|}w_{p}+\sum_{p=1}^{|r_{1}^{a}\cap r_{2}^{c}|}w_{p}+\sum_{p=1}^{|r_{1}^{c}\cap r_{2}^{a}|}w_{p}\right)}{\left(\sum_{p=1}^{|r_{1}^{a}\cup r_{2}^{a}|}w_{p}+\sum_{p=1}^{|r_{1}^{c}\cup r_{2}^{c}|}w_{p}+\sum_{p=1}^{|r_{1}^{a}\cup r_{2}^{c}|}w_{p}+\sum_{p=1}^{|r_{1}^{c}\cup r_{2}^{a}|}w_{p}\right)},$$

*where $w_{p}$ refers to the corresponding weight of the p-th common gene between the two rules.*

**Definition** **2.** *The weighted cosine measure between the two rules was defined as the ratio of the dot product of two weighted rule-vectors (i.e., $\vec{W_{1}}$ for $r_{1}$, and $\vec{W_{2}}$ for $r_{2}$) to the multiplication of the length of the two rule-vectors (Equation (8)).*

(8) $$WCos\left(r_{1},r_{2}\right)=\frac{\left(\vec{W_{1}}\cdot \vec{W_{2}}\right)}{\left|\right|\vec{W_{1}}\left||*|\right|\vec{W_{2}}\left|\right|},$$

*where $\vec{W_{1}}=\left\{u_{1},u_{2},\ldots ,u_{n},u_{n+1},u_{n+2},\ldots ,u_{2n}\right\}$ and $\vec{W_{2}}=\left\{v_{1},v_{2},\ldots ,v_{n},v_{n+1},v_{n+2},\ldots ,v_{2n}\right\}$.*

Here, the weight of genes for the first half of each vector was for the antecedent of the corresponding rule, and the weight of genes for the second half of the vector was for the consequent of the rule; see Equations (9)–(12):

(9) $$u_{j}=\left\{\begin{array}{cc} w_{i},\left(1\leq i\leq n,j=i\right), & \mathrm{if}\;g_{i}\;\mathrm{is in LHS of}\;r_{1}\\ 0, & \mathrm{if}\;g_{i}\;\mathrm{is not in LHS of}\;r_{1} \end{array}\right\}.$$

(10) $$u_{j}=\left\{\begin{array}{cc} w_{i},\left(1\leq i\leq n,j=n+i\right), & \mathrm{if}\;g_{i}\;\mathrm{is in RHS of}\;r_{1}\\ 0, & \mathrm{if}\;g_{i}\;\mathrm{is not in RHS of}\;r_{1} \end{array}\right\}.$$

(11) $$v_{j}=\left\{\begin{array}{cc} w_{i},\left(1\leq i\leq n,j=i\right), & \mathrm{if}\;g_{i}\;\mathrm{is in LHS of}\;r_{2}\\ 0, & \mathrm{if}\;g_{i}\;\mathrm{is not in LHS of}\;r_{2} \end{array}\right\}.$$

(12) $$v_{j}=\left\{\begin{array}{cc} w_{i},\left(1\leq i\leq n,j=n+i\right), & \mathrm{if}\;g_{i}\;\mathrm{is in RHS of}\;r_{2}\\ 0, & \mathrm{if}\;g_{i}\;\mathrm{is not in RHS of}\;r_{2} \end{array}\right\}.$$

Now, the proposed weighted cosine measure between the two rules could also be defined as follows:

(13) $$WCos\left(r_{1},r_{2}\right)=\frac{\sum_{i=1}^{2n}\left(u_{i}*v_{i}\right)}{\sqrt{\sum_{i=1}^{2n}{\left(u_{i}\right)}^{2}}\sqrt{\sum_{i=1}^{2n}{\left(v_{i}\right)}^{2}}}.$$

To compute the proposed weighted cosine measure, the weight of a common gene was only considered when the gene existed on the same side of both rules (see Equations (9)–(12)).

Several examples of calculating these two weighted measures are demonstrated in Supplementary File Figure S15.

#### 2.2.7. Clustering Rules Using Proposed Weighted Rule-Based Similarity Measures

After identifying the association rules, the similarity values among the pairwise resultant rules using the proposed weighted rule-based similarity measures (viz., Wζ and WCos) were computed. Thereafter, these values for each pair of rules have been integrated through the following:

(14) $$Int\_W\zeta Cos\left(r_{1},r_{2}\right)=\left(e_{1}*W\zeta \left(r_{1},r_{2}\right)+e_{2}*WCos\left(r_{1},r_{2}\right)\right)$$

where $Int\_W\zeta Cos(r_{1},r_{2})$ denotes the integrated similarity value for the rules, $r_{1}$ and $r_{2}$; whereas $e_{1}$ and $e_{2}$ are two user-defined weights for the Wζ and WCos, respectively, of the rule-pair. Because the same priorities were needed for both individual measures, $e_{1}=0.5$ and $e_{2}=0.5$. Then, we computed the corresponding dissimilarity value (i.e., dissim) from each Int_WζCos value; and finally applied the average linkage clustering method to dissim values in order to group similar rules.

(15) $$dissim\left(r_{1},r_{2}\right)=\left(1-Int\_W\zeta Cos\left(r_{1},r_{2}\right)\right).$$

#### 2.2.8. Discovery of Condensed Gene Expression Markers

A dendrogram was obtained using average linkage clustering. Dynamic tree cut method using color thresholding [24,25] was then utilized on the dendrogram in order to obtain co-expressed rule-modules. Thereafter, we computed the frequency of the geneset of each side (antecedent as well as consequent) of every rule belonging to the same rule-module, and identified the most frequent geneset from antecedent along with the most frequent geneset from consequent for each rule-module. Then, we listed all resultant genesets and ranked them by their frequency in their corresponding module from cases highest to lowest. Condensed genesets were identified, and thereafter gene expression markers were determined from the above genesets.

The condensed gene expression marker is defined as the condensed frequent expression genesets that follow “geneset equivalent pruning regulations”. These are mentioned in the following:

(i) *Geneset Equivalent Pruning regulation 1:* If there are some genesets whose genes overlapped each other (e.g., “a” and “a, b”), and if each of the genesets had the same frequency, then we considered only the geneset (e.g., “a, b”) that covered all the participating genes belonging to all these genesets, and subsequently the remaining genesets (e.g., “a”) were eliminated from the list.
(ii) *Geneset Equivalent Pruning regulation 2:* If there are some genesets whose genes overlapped each other (e.g., “a” and “a, b”) and if each of the genesets contained a different frequency, then we considered only the geneset among them which had highest frequency, and subsequently the remaining genesets were omitted.

Hence, the list of evolved condensed markers consists of both singular and complex markers depending upon corresponding condensed genesets in either antecedent or consequent of the rules of the resultant modules.

**Figure 2:**
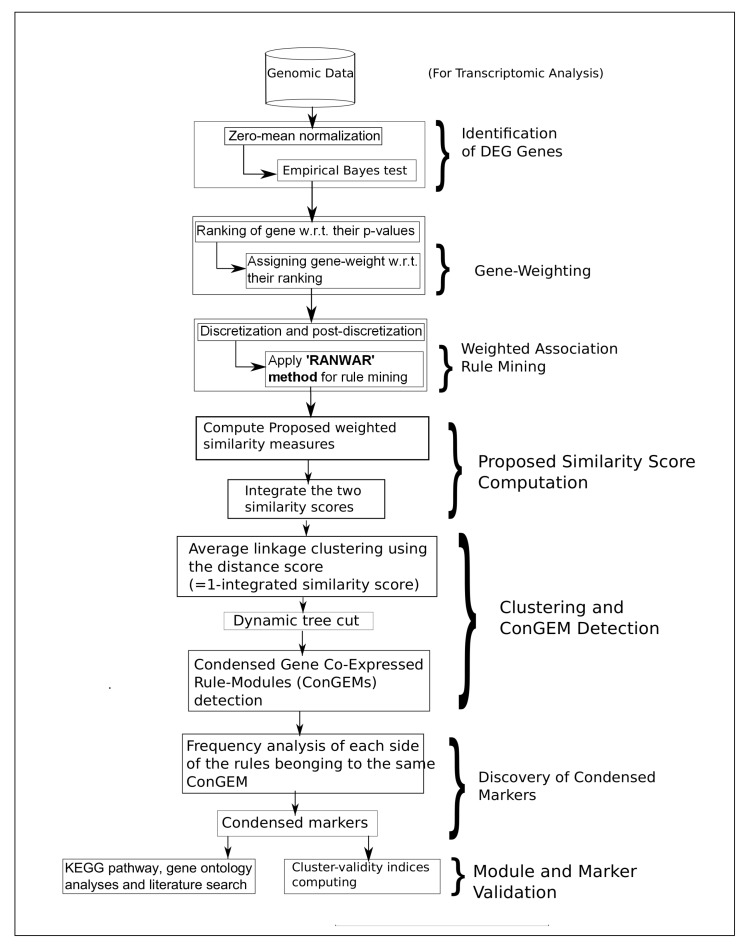
Flowchart of the proposed method, whereas “w.r.t.” denotes “with respect to”.

However, the top markers were then identified. We then performed a literature search as well as KEGG pathway and Gene Ontology enrichment analyses using the “Enrichr” database [33] for validating the resultant markers. If any related literature evidence or any related KEGG pathway or GO-term or both indicating the involvement between the disease and each participating gene belonging to any marker were found, the marker was treated as “existing”; whereas, if no literature evidence or pathway or GO-term supporting the association between the disease and any participating gene of a marker was obtained, the marker was treated as “novel”. Of note, two important articles related to disease-specific pathway extraction are [34,35]. Finally, the steps of the proposed algorithm are represented in Figure 2 for better visualization.

## 3. Results and Discussion

In this section, we first describe the source of the LUSC dataset and then provide the experimental results and discussion.

### 3.1. Dataset Information

In this paper, we utilize an LUSC dataset (GEO accession ID: GSE10245) [36] that consists of eighteen squamous cell carcinoma (SCC) samples as diseased/experimental samples and forty adenocarcinoma (AC) samples as control samples. The dataset consists of a total of 42,450 transcripts.

In addition, we apply a genome-wide DNA methylation profile of uterine cervical carcinogenesis (GEO accession ID: GSE30760) [37] having 63 cancerous uterine cervix samples (experimental samples) and 152 normal uterine cervix samples (control samples). The dataset contains a total of 27,578 transcripts.

### 3.2. Experimental Results and Discussion

In the first dataset, the genes with lowest variance were discarded. The data of the remaining genes (29,715 genes) was scaled using zero-mean normalization, and then the Limma package for performing the empirical Bayes test was applied. Subsequently, we considered the fold change of each gene along with the *p*-value provided by the Limma method. In this regard, we set 0.05 as the *p*-value cutoff, 1.4 as the upper fold change threshold, and 0.7 as the lower fold change threshold. We chose only the gene-probe that had the lowest *p*-value among all the probes for each gene, and then eliminated the other (redundant) probes of each gene for our experiment. Thereafter, we computed the Benjamini–Hochberg corrected *p*-value for each gene, and identified the genes that had corrected *p*-value less than 0.05. In this way, we obtained 316 up-regulated ($DEG_{up}$) genes and 170 down-regulated ($DEG_{down}$) genes after filtering the corrected *p*-value, resulting in a total of 486 DEGs. We ranked the resultant genes according to their corrected *p*-values. Thereafter, we generated the association rules by applying the RANWAR association rule mining technique [10] to the top hundred genes using thresholds min_wsupp = 0.50 and min_wconf = 0.70. After that, we utilized the statistics as mentioned in [38] regarding the distribution of their support and confidence, and produced the dynamic minimum support and minimum confidence thresholds for each generated rule individually using that. Next, we ranked the rules with respect to the two consecutive labels, weighted support score, and weighted confidence score such that all rules would ultimately be ranked based upon weighted confidence score in descending order, whereas in the case where more than one rule had the same confidence, those rules were also ranked by weighted support score in descending order. After that, Wζ, WCos, and Int_WζCos were computed among the pair-wise dynamically selected resultant rules. We then calculated the corresponding dissimilarity value (i.e., dissim) from each Int_WζCos value, and then utilized the average linkage clustering technique using dissim values. Thereafter, dynamic tree cut methodology was utilized on the dendrogram obtained by the average linkage clustering method for extracting the condensed gene co-expressed rule-modules (ConGEMs). Supplementary Figure S9 depicts the rule-modules through dynamic tree cut method based on computed dissim scores for the rules of the LUSC dataset using the proposed method. We obtained 44 rule-modules using the dynamic tree cut method. Each module is represented by a specific color (Figure S9). The most frequent geneset from antecedent as well as the most frequent geneset from consequent for each rule-module was identified. Condensed markers through “geneset equivalent pruning regulations” were identified from the resultant genesets. The top ten condensed markers for the LUSC dataset with their corresponding module name and their status are summarized in Table 1. Among the top ten condensed markers, we found nine (viz., {DST−}, {TP63−}, {BNC1−}, {CLCA2−}, {GJB5−}, {DSC3−,KRT5−}, {CGN+,DSC3−}, {KRT5−,NTRK2−}, and {CGN+,KRT5−}) as “existing”, while one ({DSC3−,TMEM40−,NTRK2−}) was novel. Table 2 summarizes the biological validation of the participating genes belonging to the top ten condensed markers.

DST- was the topmost condensed marker with highest frequency. DST- belonged to the consequent portion of the Purple module. The *p*-value by Limma method is 9.26 $\times \;10^{-10}$. We found some literature evidence [39,40] supporting the association between the gene and the LUSC. According to the literature [39], a significant change in the transcriptomic pattern of DST in the diseased sample-group over the normal sample-group (with significant *p*-value) was found. It is also associated with cytoskeletal structure. DST was found to be a biomarker (having significant *p*-value) in [40]. The involvement of DST with biological processes “cell adhesion” (GO: 0007155) and “epidermis development” (GO: 0008544) is also mentioned in [40]. In addition, DST is involved significantly in several LUSC-related GO-terms; for example, GO:BPs of response to wounding (*p*-value = 0.005237) [42], extracellular matrix organization (*p*-value = 0.006151) and extracellular structure organization (*p*-value = 0.006327) [42], GO:CCs of extracellular vesicular exosome (*p*-value = 1.02 $\times \;10^{-7}$), extracellular matrix part (*p*-value = 0.001283) [42], and GO:MF of calcium ion binding (*p*-value = 8.51 $\times \;10^{-10}$). Of note, here the association between the GO:BP of response to wounding and the disease LUSC is highlighted in [42]. Similarly, the relationship between the GO:BP of extracellular structure organization and the disease LUSC is also depicted in [42]. TP63− is the second most prominent condensed marker, having the second-highest frequency. It falls in the consequent portion of the Blue module as well as the consequent portion of the Brown module. Its *p*-value in the Limma method is 1.27 $\times \;10^{-10}$. The literature evidence that proves the association between TP63 and the disease LUSC is represented in [40]. Additionally, TP63 is associated with several LUSC-related GO-terms—viz., GO:BPs of regulation of the Notch signaling pathway (*p*-value = 0.024357) [43] and positive regulation of the Notch signaling pathway (*p*-value = 0.040422) [43], GO:MF of RNA polymerase II transcription regulatory region sequence-specific DNA binding transcription factor activity involved in positive regulation of transcription (*p*-value = 0.036160). The next top ranked markers are {BNC1−}, {CLCA2−}, {GJB5−}, {DSC3−,KRT5−}, {CGN+,DSC3−}, {KRT5−,NTRK2−}, {CGN+,KRT5−}, and {DSC3−,TMEM40−,NTRK2−}, respectively. The connection between BNC1/GJB5 and LUSC is highlighted in [40], whereas a similar association between CLCA2 and LUSC was found in [2,3,4,40,41,44,45]. Furthermore, BNC1 is connected with the disease-related GO:BP of response to wounding (*p*-value = 0.005237) [42]. CLCA2 is connected with the related KEGG pathways and GO-terms; for example, the KEGG pathway of Pancreatic secretion Homo sapiens hsa04972 (*p*-value = 0.020269), Renin secretion Homo sapiens hsa04924 (*p*-value = 0.037037), GO:CC of extracellular region (*p*-value = 0.000225) [42].

In the case of {DSC3−,KRT5−}, its first participating gene DSC3 is connected with the LUSC through some literature evidence [40,41,45] and some related GO-terms: viz., GO:BPs of cell–cell adhesion via plasma-membrane adhesion molecules (*p*-value = 1.15978 $\times \;10^{-10}$) and cell–cell adhesion (*p*-value = 1.28951 $\times \;10^{-10}$), GO:CCs of extracellular region (*p*-value = 0.000225) [42] and cell–cell junction (*p*-value = 0.000681), and GO:MF of calcium ion binding (*p*-value = 8.51 $\times \;10^{-10}$). Its second participating gene KRT5 is associated with LUSC through some literature evidence [40,41] and some related GO-terms: i.e., GO:BPs of regulation of Rac GTPase activity (*p*-value = 0.020821) and positive regulation of neuron projection development (*p*-value = 0.031048), and GO:CC of extracellular vesicular exosome (*p*-value = 1.02 $\times \;10^{-7}$). Similarly, for {CGN+,DSC3−}, its first participating gene CGN is linked in the SCC through literature evidence [46] and some pathway and GO-term information, such as the KEGG pathway of tight junction Homo sapiens hsa04530 (*p*-value = 0.04347487), and GO:CC of cell–cell junction (*p*-value = 0.000681). Its second participating gene DSC3 is connected with LUSC through [40,41,45] and some related GO-terms: i.e., GO:BPs of cell–cell adhesion via plasma-membrane adhesion molecules (*p*-value = 1.15978 $\times \;10^{-10}$) and cell–cell adhesion (*p*-value = 1.28951 $\times \;10^{-10}$), GO:CCs of extracellular region (*p*-value = 0.000225) [42] and cell–cell junction (*p*-value = 0.000681), and GO:MF of calcium ion binding (*p*-value = 8.51 $\times \;10^{-10}$). However, similar related information for the next ranked condensed markers are demonstrated in Table 1 and Table 2.

Notably, the tenth ranked marker (namely, {DSC3−,TMEM40−,NTRK2−}) was the only novel marker found in the antecedent part of the module in yellow. In this case, the activities of these two genes (DSC3 and NTRK2) in LUSC are available in the literature and are supported by various related GO-terms (in Table 2). However, the potential role of TMEM40 is not clear from either our literature search or pathway/GO analysis. Overall, {DSC3−,TMEM40−,NTRK2−} as a geneset might serve as a novel marker. Considering that the majority of the top ten markers were known markers, this new marker ({DSC3−,TMEM40−,NTRK2−}) may be promising and warrant future investigation.

We further compared our method with the method by Su and Pan [40] in Table 2. Interestingly, most of the genes in Table 2 obtained by our proposed method (i.e., eight out of ten genes) namely DST, TP63, BNC1, CLCA2, GJB5, DSC3, KRT5 and NTRK2 overlapped the list of marker genes in [40]. Two genes, CGN and TMEM40 were not found in [40], but the association of the disease with CGN is identified by Molina-Pinelo et al. [46]. In the case of pathway and GO enrichment studies, we considered only those KEGG pathways and GO-terms found in the literature that were potentially associated with the disease. For example, the gene DST is linked to SCC through some disease-related significant GO:BP terms—namely, extracellular structure organization (GO:0043062) (*p*-value = 0.006327), response to wounding (GO:0009611) (*p*-value = 0.005237), and some disease-related significant GO:CC terms such as extracellular matrix part (GO:0044420) (*p*-value = 0.001283). All such information is supported by Ge et al. [42]. On the other hand, Su and Pan [40] mentioned all these significant GO terms, but they did not provide specific literature evidence. Of note, most of the GO terms mentioned in Table 2 obtained by our method were distinct from the identified GO terms obtained by Su and Pan [40].

In addition, we provided a comparative study of several well-known internal cluster-validity indices (e.g., average Dunn index (avgDI), average Silhoutte width (avgSW), average scaled connectivity (avgSC), average cluster coefficient (avgCC), average maximum adjacency ratio (avgMAR), density, and centralization (Ctlz)) as well as two external cluster-validity indices (Rand index and adjusted Rand index) between our proposed rule-module discovery method and the traditional gene-module discovery methods using several existing similarity (connectivity) measures, such as weighted TOM using Pearson’s correlation co-efficient (i.e., wTOM[pcc]), weighted TOM using Spearman’s correlation (i.e., wTOM[sc]), generalized TOM of degree 0 using Pearson’s correlation co-efficient (i.e., GTOM0[pcc]), generalized TOM of degree 0 using Spearman’s correlation (i.e., GTOM0[sc]), generalized TOM of degree 1 using Pearson’s correlation co-efficient (i.e., GTOM1[pcc]), generalized TOM of degree 1 using Spearman’s correlation (i.e., GTOM1[sc]), generalized TOM of degree 2 using Pearson’s correlation co-efficient (i.e., GTOM2[pcc]), generalized TOM of degree 2 using Spearman’s correlation (i.e., GTOM2[sc]), generalized TOM of degree 3 using Pearson’s correlation co-efficient (i.e., GTOM3[pcc]), and generalized TOM of degree 3 using Spearman’s correlation (i.e., GTOM3[sc]). Of note, we also collected the actual class-labels of the features from the partitioning around medoids method [47] by fixing the number of clusters equal to the number of the modules produced in the dynamic tree cut method.

According to Table 3, there were nine cluster validity indices, each denoting each of the nine cases. In Table 3, each row stands for each individual validity index. Here, we identified the best score (bold font) as well as the best method among all the methods (i.e., a total of eleven methods) for each specific cluster validity index (denoting each row of Table 3. For example, for the validity index, namely average clustering coefficient (avgCC), 2.53 $\times \;10^{-1}$ was the best score among all the other scores at that row avgCC in Table 3. Thus, the corresponding method (i.e., proposed method here) was considered the best method for the validity index avgCC. Similarly, for another validity index (namely centralization), 1.36 $\times \;10^{-1}$ was the best score among all the scores at that row representing the centralization, and wTOM[pcc] was the best scorer for the validity index centralization. Following this approach, we obtained five such cases (i.e., five cluster validity indices, namely avgDI, avgCC, avgMAR, Density, and Rand index) for which our method generated best score. There were four remaining cases or validity indices, among which wTOM[pcc] produced best score for two of them (i.e., avgSW and centralization), and GTOM1[sc] yielded the best score for one of the remaining cases (i.e., avgSC), whereas all methods failed to produce values for the remaining case (i.e., adjusted Rand index). Furthermore, “win-draw-loss” was considered and computed for these methods in a pairwise manner. For example, our method won (i.e., produced better outcome) over each of the methods—namely wTOM[sc], GTOM0[sc], GTOM1[pcc], GTOM1[sc], GTOM2[sc], and GTOM3[sc]—for seven times, whereas our method lost (i.e., produced worse outcome) over each of these six methods only once. Furthermore, the proposed method provided five wins and three losses when compared to wTOM[pcc], whereas our method produced six wins and two losses over each of GTOM0[pcc], GTOM2[pcc], and GTOM3[pcc]. Details are shown in the first row of the summary table (Table 9), in which the number of wins, draws, and losses of our proposed method (denoted in rows) over each of the other methods (represented in columns) is mentioned. Overall, our method outperformed the other methods. The dendrograms and the plots of Silhouette width are provided in the Supplementary Files (Figures S1–S12).

Similar to the first dataset, we applied our proposed method to the second dataset (uterine cervical carcinogenesis dataset). In this case, we first obtained 9024 hyper-methylated and 4185 hypo-methylated genes by Limma analysis followed by Benjamini–Hochberg *p*-value multiple test correction. We ran the RANWAR tool for generating association rules with the Wsupp = 0.50 and WConf = 0.70. After that, like the first dataset, we also computed the (dynamic) minimum support and minimum confidence thresholds for each generated rule separately using the data-distributional theory proposed in [38]. Using these dynamically selected rules, we computed the integrated similarity scores using the proposed weighted Jaccard and weighted Cosine measure. The corresponding distance was then calculated from the integrated similarity measure. Using the distance, we performed average linkage clustering and dynamic tree cut, respectively. Of note, we also collected the actual class-labels of the features from the partitioning around medoids method [47] by fixing the number of clusters equal to the number of the modules produced in the dynamic tree cut method. As a result, our method led to a total of 21 rule-modules. In Supplementary Figure S14, these rule-modules were labeled by color: black, blue, brown, cyan, dark red, green, green-yellow, grey60, light cyan, light green, light yellow, magenta, midnight blue, pink, purple, red, royal blue, salmon, tan, turquoise, and yellow, which had 55, 66, 59, 40, 37, 57, 43, 39, 39, 38, 38, 45, 40, 55, 45, 57, 37, 42, 43, 66, and 59 participating rules, respectively. None of the existing methods could generate a sufficient number of clusters for this dataset: all these methods (see below) either generated a single cluster or no cluster. For example, each of these methods—wTOM[pcc], wTOM[sc], GTOM0[pcc], GTOM0[sc], GTOM1[pcc], GTOM2[pcc] and GTOM3[pcc]—extracted single cluster colored turquoise, whereas all the remaining methods (GTOM1[sc], GTOM2[sc], and GTOM3[sc]) could not generate any cluster. In terms of performance, our method had better performance over eight others (wTOM[sc], GTOM0[sc], GTOM1[pcc], GTOM1[sc], GTOM2[pcc], GTOM2[sc], GTOM3[pcc], and GTOM3[sc]) out of the ten existing methods when we measured by the number of wins, whereas the overall performance of our method was same as the remaining two methods (wTOM[pcc] and GTOM0[pcc]) as measured by the numbers of wins and losses. Specifically, the number of “win-draw-loss” of our method over wTOM[sc], GTOM0[sc], GTOM1[pcc], GTOM1[sc], GTOM2[pcc], GTOM2[sc], GTOM3[pcc], and GTOM3[sc] was 5-1-3, 6-1-2, 5-1-3, 7-1-1, 5-1-3, 7-1-1, 5-1-3, and 7-1-1, respectively, whereas both of the remaining methods (wTOM[pcc] and GTOM0[pcc]) had 4-1-4. For the second dataset, the detailed comparative results of the different cluster-validity index measures between our proposed method and the other methods were represented in Table 4. Since we obtained more wins overall in the majority of cases for both datasets, our method was better than any other existing methods.

In addition, we included a simulation study for each of the datasets, and also compared the performance of our method and the other methods. For this purpose, we first picked up fifty percent features (genes or rules) from the above resultant set of dynamic rules. Thereafter, we calculated the integrated similarity scores using the proposed weighted Jaccard and weighted Cosine measure. The respective distance was then computed from the integrated similarity measure. Using the distance, we carried out average linkage clustering and dynamic tree cut, respectively, as we did previously. Then the resultant modules were identified. Of note, we also collected the actual class-labels of the features from the partitioning around medoids method [47] by fixing the number of clusters equal to the number of the modules produced in the dynamic tree cut method. Thereafter, we computed the previously mentioned internal cluster validity indices as well as the two external validity indices. For the LUSC dataset, the number of “win-draw-loss” of our method over wTOM[pcc], wTOM[sc], GTOM0[pcc], GTOM0[sc], GTOM1[pcc], GTOM1[sc], GTOM2[pcc], GTOM2[sc], GTOM3[pcc], and GTOM3[sc] in the first simulation was 5-0-4, 7-0-2, 6-0-3, 7-0-2, 5-0-4, 5-0-4, 5-0-4, 6-0-3, 6-0-3, and 5-0-4, respectively, whereas the same for the second simulation was 6-0-3, 8-0-1, 7-0-2, 8-0-1, 7-0-2, 8-0-1, 7-0-2, 8-0-1, 7-0-2, and 8-0-1, respectively. For the uterine cervical carcinogenesis, this number for the first simulation was 5-0-4, 5-0-4, 6-0-3, 6-0-3, 6-0-3, 8-0-1, 6-0-3, 7-0-2, 6-0-3, and 7-0-2, respectively, whereas the number for the second simulation was 5-0-4, 6-0-3, 6-0-3, 6-0-3, 6-0-3, 7-0-2, 6-0-3, 7-0-2, 6-0-3, and 7-0-2, respectively. Furthermore, the details of cluster validity indices in the four simulation studies are depicted in Table 5, Table 6, Table 7 and Table 8, respectively. The summary tables containing “win-draw-loss” information for all the datasets are represented in Table 9.

The noteworthy difference between our proposed method and an existing technique developed by Sethi et al. [32] is described in the following. Sethi et al. selected the non-redundant features (genes) using statistical impurity-based measures (i.e., Gini Index, Max Minority, and Twoing rule measures), whereas in our proposed method we performed a well-known statistical hypothesis test—the empirical Bayes test using the Limma package [22]—that was applied to determine the (non-redundant) differentially expressed genes. In Limma, the null hypothesis is that “there is no difference between the means of the two groups (diseased group and normal group)”. Since differentially expressed genes identified by Limma make more sense in gene-based rule-clustering or in the characterization of disease than the selected genes through the impurity-based measures, our method is more advantageous than the other. In other words, there are both differentially expressed genes and non-differentially expressed genes present in the resulting rules by [32], whereas our method produces only differentially expressed genes, and no non-differentially expressed genes. The second advantage of our method is that Sethi et al. provides only the rules of genes, whereas our method provides the rules of genes with their status of differential expression (either up-regulated or down-regulated).

In addition, we slightly modified the two weighted similarity measures (Jaccard and Cosine). In the numerator of the weighted Jaccard measure made by Sethi et al., they counted the number of common genes either on the same side of both the rules (i.e., LHS or RHS of both the rules) or on the opposite sides (i.e., LHS of one rule and RHS of the other rule) and then added 1 with each count, separately, then multiplied each resultant value with the weighted-sum of the common genes of each combination of LHS and RHS of the two rules, and finally summed the weighted-sum of the four combinations of LHS and RHS of the two rules [32]. The addition of 1 to each count (the number of common genes) for each of four combinations of LHS and RHS of the two rules is redundant, which was not done in case of the numerator of the weighted Jaccard measure developed by us. In our case, we determined the common genes either on the same side of both the rules (i.e., LHS or RHS of both the rules) or on the opposite sides (i.e., LHS of one rule and RHS of the other rule), and thereafter simply computed the weighted-sum of the common genes of each combination of LHS and RHS of the two rules. In the denominator of the weighted Jaccard measure made by Sethi et al., they added an addition of the total weights of all the present genes of both rules. However, in the denominator of the weighted Jaccard measure developed by us, we computed the weighted sum of the union of genes existing either on the same side of both the rules or on the opposite sides. Additionally, the weighted vectors of weighted Cosine measure are different for our proposed method and the method by Sethi et al. [32].

We used R scripts for statistical tests and gene-module detection, and wrote in-house codes by MatLab for computing integrated similarity measure. The codes are available upon request.

## 4. Conclusions

There are many bioinformatics approaches as well as tools for genetic marker discovery. Genetic-rule-module approach—which can enhance the discovery of the interaction mechanisms of the genes in disease phenotypes—has not been applied yet. Hence, in this article, we introduced two new rule-based similarity measures—weighted rank-based Jaccard and Cosine measures—to obtain similarity scores between pairwise rules. Based on that, we proposed a new computational framework for identifying condensed gene co-expression modules (ConGEMs). In addition, we detected the condensed markers from the resultant co-expressed rule-modules. Our application to a real gene expression dataset for LUSC and a real methylation dataset for uterine cervical carcinogenesis demonstrated this method to be effective. In LUSC analysis, we obtained a total of 44 rule-modules (ConGEMs) from the dataset, from which we assessed the corresponding top condensed markers. Our evaluation of these markers through literature, KEGG pathway, and GO terms suggested that our results were reliable and likely useful. Specifically, we found a new marker {DSC3−,TMEM40−,NTRK2−}, which is promising based on gene function and related studies. Moreover, a comparative study between our proposed rule-module discovery method and the traditional gene-module discovery methods using several existing similarity (connectivity) measures based upon several well-known cluster validity indices are also provided. In summary, our method will be useful for identifying rule-modules along with respective markers from many available genome-based gene expression datasets, and can be applied to RNA-seq or related expression data.

In addition, there are a couple of related directions that are required to further enhance our current method to solve more research objectives. Among them, finding significant associations through graphical model is important. One such method was developed by Scutari and Nagarajan [48]. Other interesting research problems are the impact of noise in biomolecular network or genetic module on the pairwise dependencies, as well as the conditional dependencies through various motif-based studies [49], and the functional impact of identifying dense subgraphs in biological network or genetic module [50]. Since rule-based module discovery has rarely been attempted prior to our work, as future work, we will design an improved version of the current method using the aforementioned three research objectives in multi-omics cancer datasets.

## Figures and Tables

**Table 1:** Top ten condensed markers (CGMs) for the lung squamous cell carcinoma (LUSC) dataset.

Rank	Condensed Marker (CGM)	Module Label	Availability of Biological Evidence	Status of Condensed Marker
1	DST-	purple (consequent)	Available	**Existing**
2	TP63-	blue, brown (consequent)	Available	**Existing**
3	BNC1-	pink (consequent)	Available	**Existing**
4	CLCA2-	yellow (consequent)	Available	**Existing**
5	GJB5-	dark red (consequent)	Available	**Existing**
6	{DSC3-, KRT5-}	dark turquoise (antecedent)	Available for both	**Existing**
7	{CGN+, DSC3-}	salmon (antecedent)	Available for both	**Existing**
8	{KRT5-, NTRK2-}	blue (antecedent)	Available for both	**Existing**
9	{CGN+, KRT5-}	light green (antecedent)	Available for both	**Existing**
10	{DSC3-, TMEM40-, NTRK2-}	yellow (antecedent)	Available for DSC3 and NTRK2, not found for TMEM40	**Novel**

**Table 2:** Biological validations of individual genes belonging to the CGMs in Table 1 for the LUSC dataset.

Individual Gene	*p*-Value	Literature Evidence	KEGG Pathway and GO-Terms (*p*-Value)
DST	9.26 $\times \;10^{-10}$	[39,40,41]	**GO:BPs:** response to wounding (GO:0009611) (*p*-value = 0.005237) [42], extracellular matrix organization (GO:0030198) (*p*-value = 0.006151), extracellular structure organization (GO:0043062) (*p*-value = 0.006327) [42]; **GO:CCs:** extracellular vesicular exosome (GO:0070062) (*p*-value = 1.02 $\times \;10^{-7}$), extracellular matrix part (GO:0044420) (*p*-value = 0.001283) [42]; **GO:MF:** calcium ion binding (GO:0005509) (8.51 $\times \;10^{-10}$).
TP63	1.27 $\times \;10^{-10}$	[40]	**GO:BPs:** regulation of Notch signaling pathway (GO:0008593) (*p*-value = 0.024357) [43], positive regulation of Notch signaling pathway (GO:0045747) (*p*-value = 0.040422) [43]; **GO:MF:** RNA polymerase II transcription regulatory region sequence-specific DNA binding transcription factor activity involved in positive regulation of transcription (GO:0001228) (*p*-value = 0.036160).
BNC1	2.82 $\times \;10^{-9}$	[40]	**GO:BP:** response to wounding (GO:0009611) (*p*-value = 0.005237) [42].
CLCA2	1.28 $\times \;10^{-10}$	[2,3,4,40,41,44,45]	**KEGG pathways:** Pancreatic secretion_Homo sapiens_hsa04972 (*p*-value = 0.020269), Renin secretion_Homo sapiens_hsa04924 (*p*-value = 0.037037); **GO:CC:** extracellular region (GO:0005576) (*p*-value = 0.000225) [42].
GJB5	1.94 $\times \;10^{-10}$	[40]	
CGN	1.96 $\times \;10^{-10}$	[46]	**KEGG pathway:** Tight junction_Homo sapiens_hsa04530 (*p*-value = 0.04347487); **GO:CC:** cell-cell junction (GO:0005911) (*p*-value = 0.000681).
DSC3	3.08 $\times \;10^{-11}$	[40,41,45]	**GO:BPs:** cell–cell adhesion via plasma-membrane adhesion molecules (GO:0098742) (*p*-value = 1.15978 $\times \;10^{-10}$), cell–cell adhesion (GO:0098609) (*p*-value = 1.28951 $\times \;10^{-10}$); **GO:CCs:** extracellular region (GO:0005576) (*p*-value = 0.000225) [42], cell–cell junction (GO:0005911) (*p*-value = 0.000681); **GO:MF:** calcium ion binding (GO:0005509) (*p*-value = 8.51 $\times \;10^{-10}$).
KRT5	6.50 $\times \;10^{-10}$	[40,41]	**GO:BP:** regulation of Rac GTPase activity (GO:0032314) (*p*-value = 0.020821), positive regulation of neuron projection development (GO:0010976) (*p*-value = 0.031048); **GO:CC:** extracellular vesicular exosome (GO:0070062) (*p*-value = 1.02 $\times \;10^{-7}$).
NTRK2	1.47 $\times \;10^{-9}$	[40]	**GO:BPs:** regulation of Rac GTPase activity (GO:0032314) (*p*-value = 0.020821), positive regulation of neuron projection development (GO:0010976) (*p*-value = 0.031048); **GO:MF:** growth factor binding (GO:0019838) (*p*-value = 0.011462) [42].
TMEM40	1.29 $\times \;10^{-9}$	-	

“GO” denotes Gene-Ontology.

**Table 3:** Comparison of proposed rule-based gene-module detection method and other existing geneset-based gene-module detection methods for the LUSC dataset.

Validty Index	Proposed	wTOM[pcc]	wTOM[sc]	GTOM0[pcc]	GTOM0[sc]	GTOM1[pcc]	GTOM1[sc]	GTOM2[pcc]	GTOM2[sc]	GTOM3[pcc]	GTOM3[sc]
**avgDI ⇑**	**3.82** $\times \;10^{-1}$	Inf	Inf	Inf	Inf	-	-	-	-	-	-
**avgSW ⇑**	7.82 $\times \;10^{-2}$	**9.97** $\times \;10^{-2}$	3.94 $\times \;10^{-2}$	6.55 $\times \;10^{-2}$	2.89 $\times \;10^{-2}$	-	-	-	-	-	-
**avgSC ⇓**	6.80 $\times \;10^{-1}$	4.24 $\times \;10^{-1}$	3.41 $\times \;10^{-1}$	3.39 $\times \;10^{-1}$	3.02 $\times \;10^{-1}$	9.41 $\times \;10^{-2}$	**6.3** $\times \;10^{-2}$	1.66 $\times \;10^{-1}$	6.53 $\times \;10^{-2}$	1.65 $\times \;10^{-1}$	6.53 $\times \;10^{-2}$
**avgCC ⇑**	**2.53** $\times \;10^{-1}$	1.52 $\times \;10^{-1}$	8.74 $\times \;10^{-2}$	1.30 $\times \;10^{-1}$	8.64 $\times \;10^{-2}$	8.86 $\times \;10^{-2}$	5.18 $\times \;10^{-2}$	1.47 $\times \;10^{-1}$	5.38 $\times \;10^{-2}$	1.47 $\times \;10^{-1}$	5.38 $\times \;10^{-2}$
**avgMAR ⇑**	**2.99** $\times \;10^{-1}$	1.26 $\times \;10^{-1}$	8.24 $\times \;10^{-2}$	1.24 $\times \;10^{-1}$	1.00 $\times \;10^{-1}$	-	-	-	-	-	-
**Density ⇑**	**2.21** $\times \;10^{-1}$	9.83 $\times \;10^{-2}$	3.99 $\times \;10^{-2}$	6.42 $\times \;10^{-2}$	2.90 $\times \;10^{-2}$	7.782 $\times \;10^{-3}$	3.18 $\times \;10^{-3}$	2.40 $\times \;10^{-2}$	3.30 $\times \;10^{-3}$	2.40 $\times \;10^{-2}$	3.30 $\times \;10^{-3}$
**Centralization ⇑**	1.04 $\times \;10^{-1}$	**1.36** $\times \;10^{-1}$	7.85 $\times \;10^{-2}$	1.28 $\times \;10^{-1}$	6.83 $\times \;10^{-2}$	7.64 $\times \;10^{-2}$	4.83 $\times \;10^{-2}$	1.24 $\times \;10^{-1}$	4.82 $\times \;10^{-2}$	1.24 $\times \;10^{-1}$	4.82 $\times \;10^{-2}$
**Rand index ⇑**	**2.21** $\times \;10^{-1}$	-	-	-	-	-	-	-	-	-	-
**Adjusted Rand index ⇑**	-	-	-	-	-	-	-	-	-	-	-

⇑ signifies that a higher value of the corresponding validity index is better in determining the gene modules, while ⇓ denotes the reverse of the above statement. For each validity index, an entry denoted with bold font indicates that the corresponding method is the best performer in terms of the corresponding index (row-wise). wTOM[pcc]: weighted TOM using Pearson’s correlation coefficient; wTOM[sc]: weighted TOM using Spearman’s correlation; GTOM0[pcc]: generalized TOM of degree 0 using Pearson’s correlation coefficient; GTOM0[sc]: generalized TOM of degree 0 using Spearman’s correlation; avgDI: average Dunn index; avgSW: average Silhoutte width; avgSC: average scaled connectivity; avgCC: average cluster coefficient; avgMAR: average maximum adjacency ratio.

**Table 4:** Comparison of proposed rule-based gene-module detection method and other existing geneset-based gene-module detection methods for the cervical carcinogenesis dataset.

Validty Index	Proposed	wTOM[pcc]	wTOM[sc]	GTOM0[pcc]	GTOM0[sc]	GTOM1[pcc]	GTOM1[sc]	GTOM2[pcc]	GTOM2[sc]	GTOM3[pcc]	GTOM3[sc]
**avgDI ⇑**	**3.47** $\times \;10^{-1}$	Inf	Inf	Inf	Inf	Inf	-	Inf	-	Inf	-
**avgSW ⇑**	1.85 $\times \;10^{-1}$	3.13 $\times \;10^{-1}$	2.12 $\times \;10^{-1}$	9.16 $\times \;10^{-1}$	1.61 $\times \;10^{-1}$	**9.90** $\times \;10^{-1}$	-	**9.90** $\times \;10^{-1}$	-	**9.90** $\times \;10^{-1}$	-
**avgSC ⇓**	7.41 $\times \;10^{-1}$	9.99 $\times \;10^{-1}$	6.39 $\times \;10^{-1}$	9.77 $\times \;10^{-1}$	5.49 $\times \;10^{-1}$	9.90 $\times \;10^{-1}$	**1.08** $\times \;10^{-1}$	9.90 $\times \;10^{-1}$	1.26 $\times \;10^{-1}$	9.90 $\times \;10^{-1}$	1.27 $\times \;10^{-1}$
**avgCC ⇑**	**2.77** $\times \;10^{-1}$	9.64 $\times \;10^{-1}$	2.76 $\times \;10^{-1}$	9.55 $\times \;10^{-1}$	2.58 $\times \;10^{-1}$	**9.90** $\times \;10^{-1}$	1.81 $\times \;10^{-1}$	**9.90** $\times \;10^{-1}$	1.98 $\times \;10^{-1}$	**9.90** $\times \;10^{-1}$	1.99 $\times \;10^{-1}$
**avgMAR ⇑**	3.25 $\times \;10^{-1}$	**9.64** $\times \;10^{-1}$	2.53 $\times \;10^{-1}$	9.52 $\times \;10^{-1}$	2.75 $\times \;10^{-1}$	-	-	-	-	-	-
**Density ⇑**	1.87 $\times \;10^{-1}$	9.64 $\times \;10^{-1}$	2.11 $\times \;10^{-1}$	9.49 $\times \;10^{-1}$	1.59 $\times \;10^{-1}$	**9.80** $\times \;10^{-1}$	1.01 $\times \;10^{-2}$	**9.80** $\times \;10^{-1}$	1.12 $\times \;10^{-2}$	**9.80** $\times \;10^{-1}$	1.26 $\times \;10^{-2}$
**Centralization ⇑**	1.26 $\times \;10^{-1}$	1.04 $\times \;10^{-2}$	1.21 $\times \;10^{-1}$	2.31 $\times \;10^{-2}$	**1.33** $\times \;10^{-1}$	1.01 $\times \;10^{-2}$	8.54 $\times \;10^{-2}$	1.01 $\times \;10^{-2}$	8.80 $\times \;10^{-2}$	1.01 $\times \;10^{-2}$	8.84 $\times \;10^{-2}$
**Rand index ⇑**	**4.29** $\times \;10^{-1}$	-	-	-	-	-	-	-	-	-	-
**Adjusted Rand index ⇑**	-	-	-	-	-	-	-	-	-	-	-

⇑ signifies that a higher value of the corresponding validity index is better in determining the gene modules, while ⇓ denotes the reverse of the above statement. For each validity index, an entry denoted with bold font indicates that the corresponding method is the best performer in terms of the corresponding index (row-wise).

**Table 5:** Comparison of proposed rule-based gene-module detection method and other existing geneset-based gene-module detection methods in a simulation study for the LUSC dataset.

Validty Index	Proposed	wTOM[pcc]	wTOM[sc]	GTOM0[pcc]	GTOM0[sc]	GTOM1[pcc]	GTOM1[sc]	GTOM2[pcc]	GTOM2[sc]	GTOM3[pcc]	GTOM3[sc]
**avgDI ⇑**	**3.82** $\times \;10^{-1}$	Inf	Inf	Inf	Inf	Inf	Inf	Inf	Inf	Inf	Inf
**avgSW ⇑**	5.91 $\times \;10^{-2}$	2.97 $\times \;10^{-1}$	-	5.50 $\times \;10^{-1}$	-	5.54 $\times \;10^{-1}$	2.80 $\times \;10^{-1}$	8.66 $\times \;10^{-1}$	4.51 $\times \;10^{-1}$	**8.96** $\times \;10^{-1}$	6.11 $\times \;10^{-1}$
**avgSC ⇓**	6.74 $\times \;10^{-1}$	9.01 $\times \;10^{-1}$	9.10 $\times \;10^{-1}$	9.10 $\times \;10^{-1}$	9.10 $\times \;10^{-1}$	6.92 $\times \;10^{-1}$	**5.01** $\times \;10^{-1}$	8.83 $\times \;10^{-1}$	6.83 $\times \;10^{-1}$	9.06 $\times \;10^{-1}$	6.81 $\times \;10^{-1}$
**avgCC ⇑**	2.51 $\times \;10^{-1}$	6.55 $\times \;10^{-1}$	5.29 $\times \;10^{-1}$	5.29 $\times \;10^{-1}$	5.29 $\times \;10^{-1}$	6.70 $\times \;10^{-1}$	5.14 $\times \;10^{-1}$	8.71 $\times \;10^{-1}$	6.16 $\times \;10^{-1}$	**8.96** $\times \;10^{-1}$	7.20 $\times \;10^{-1}$
**avgMAR ⇑**	2.98 $\times \;10^{-1}$	**6.53** $\times \;10^{-1}$	5.31 $\times \;10^{-1}$	5.31 $\times \;10^{-1}$	5.31 $\times \;10^{-1}$	-	-	-	-	-	-
**Density ⇑**	2.19 $\times \;10^{-1}$	6.50 $\times \;10^{-1}$	5.27 $\times \;10^{-1}$	5.27 $\times \;10^{-1}$	5.27 $\times \;10^{-1}$	5.09 $\times \;10^{-1}$	1.25 $\times \;10^{-1}$	7.95 $\times \;10^{-1}$	2.34 $\times \;10^{-1}$	**8.23** $\times \;10^{-1}$	3.38 $\times \;10^{-1}$
**Centralization ⇑**	**1.06** $\times \;10^{-1}$	7.41 $\times \;10^{-2}$	5.47 $\times \;10^{-2}$	5.47 $\times \;10^{-2}$	5.47 $\times \;10^{-2}$	1.01 $\times \;10^{-1}$	9.04 $\times \;10^{-2}$	1.04 $\times \;10^{-1}$	1.04 $\times \;10^{-1}$	8.89 $\times \;10^{-2}$	8.89 $\times \;10^{-2}$
**Rand index ⇑**	**7.01** $\times \;10^{-1}$	-	-	-	-	-	-	-	-	-	-
**Adjusted Rand index ⇑**	**3.53** $\times \;10^{-1}$	-	-	-	-	-	-	-	-	-	-

⇑ signifies that a higher value of the corresponding validity index is better in determining the gene modules, while ⇓ denotes the reverse of the above statement. For each validity index, an entry denoted with bold font indicates that the corresponding method is the best performer in terms of the corresponding index (row-wise).

**Table 6:** Comparison of proposed rule-based gene-module detection method and other existing geneset-based gene-module detection methods in the second simulation study for the LUSC dataset.

Validty Index	Proposed	wTOM[pcc]	wTOM[sc]	GTOM0[pcc]	GTOM0[sc]	GTOM1[pcc]	GTOM1[sc]	GTOM2[pcc]	GTOM2[sc]	GTOM3[pcc]	GTOM3[sc]
**avgDI ⇑**	**3.81** $\times \;10^{-1}$	Inf	Inf	Inf	-	-	-	-	-	-	-
**avgSW ⇑**	7.11 $\times \;10^{-2}$	**1.24** $\times \;10^{-1}$	5.51 $\times \;10^{-2}$	1.00 $\times \;10^{-1}$	-	-	-	-	-	-	-
**avgSC ⇓**	6.83 $\times \;10^{-1}$	5.50 $\times \;10^{-1}$	4.34 $\times \;10^{-1}$	4.34 $\times \;10^{-1}$	4.34 $\times \;10^{-1}$	1.51 $\times \;10^{-1}$	**9.67** $\times \;10^{-2}$	2.37 $\times \;10^{-1}$	1.18 $\times \;10^{-1}$	2.65 $\times \;10^{-1}$	1.14 $\times \;10^{-1}$
**avgCC ⇑**	**2.53** $\times \;10^{-1}$	1.96 $\times \;10^{-1}$	1.09 $\times \;10^{-1}$	1.09 $\times \;10^{-1}$	1.09 $\times \;10^{-1}$	1.42 $\times \;10^{-1}$	7.85 $\times \;10^{-2}$	1.98 $\times \;10^{-1}$	9.23 $\times \;10^{-2}$	2.39 $\times \;10^{-1}$	9.87 $\times \;10^{-2}$
**avgMAR ⇑**	**2.99** $\times \;10^{-1}$	1.71 $\times \;10^{-1}$	1.05 $\times \;10^{-1}$	1.05 $\times \;10^{-1}$	1.05 $\times \;10^{-1}$	-	-	-	-	-	-
**Density ⇑**	**2.21** $\times \;10^{-1}$	1.42 $\times \;10^{-1}$	5.99 $\times \;10^{-2}$	5.99 $\times \;10^{-2}$	5.99 $\times \;10^{-2}$	2.23 $\times \;10^{-2}$	7.90 $\times \;10^{-3}$	5.02 $\times \;10^{-2}$	1.04 $\times \;10^{-2}$	6.23 $\times \;10^{-2}$	1.09 $\times \;10^{-2}$
**Centralization ⇑**	1.03 $\times \;10^{-1}$	1.21 $\times \;10^{-1}$	8.84 $\times \;10^{-2}$	8.84 $\times \;10^{-2}$	8.84 $\times \;10^{-2}$	1.34 $\times \;10^{-1}$	7.68 $\times \;10^{-2}$	1.68 $\times \;10^{-1}$	8.13 $\times \;10^{-2}$	**1.83** $\times \;10^{-1}$	8.79 $\times \;10^{-2}$
**Rand index ⇑**	**7.18** $\times \;10^{-1}$	-	-	-	-	-	-	-	-	-	-
**Adjusted Rand index ⇑**	**1.75** $\times \;10^{-1}$	-	-	-	-	-	-	-	-	-	-

⇑ signifies that a higher value of the corresponding validity index is better in determining the gene modules, while ⇓ denotes the reverse of the above statement. For each validity index, an entry denoted with bold font indicates that the corresponding method is the best performer in terms of the corresponding index (row-wise).

**Table 7:** Comparison of proposed rule-based gene-module detection method and other existing geneset-based gene-module detection methods in the first simulation study for the cervical carcinogenesis dataset.

Validty Index	Proposed	wTOM[pcc]	wTOM[sc]	GTOM0[pcc]	GTOM0[sc]	GTOM1[pcc]	GTOM1[sc]	GTOM2[pcc]	GTOM2[sc]	GTOM3[pcc]	GTOM3[sc]
**avgDI ⇑**	**4.23** $\times \;10^{-1}$	Inf	Inf	Inf	Inf	Inf	-	Inf	-	Inf	-
**avgSW ⇑**	9.33 $\times \;10^{-2}$	3.83 $\times \;10^{-1}$	1.89 $\times \;10^{-1}$	9.07 $\times \;10^{-1}$	1.55 $\times \;10^{-1}$	**9.80 $\times \;10^{-1}$**	-	**9.80** $\times \;10^{-1}$	-	**9.80** $\times \;10^{-1}$	-
**avgSC ⇓**	7.45 $\times \;10^{-1}$	9.91 $\times \;10^{-1}$	6.20 $\times \;10^{-1}$	**1.22** $\times \;10^{-1}$	**1.22** $\times \;10^{-1}$	9.80 $\times \;10^{-1}$	**1.22** $\times \;10^{-1}$	9.80 $\times \;10^{-1}$	1.30 $\times \;10^{-1}$	9.80 $\times \;10^{-1}$	1.30 $\times \;10^{-1}$
**avgCC ⇑**	2.59 $\times \;10^{-1}$	9.60 $\times \;10^{-1}$	2.63 $\times \;10^{-1}$	1.20 $\times \;10^{-1}$	1.20 $\times \;10^{-1}$	**9.80** $\times \;10^{-1}$	1.20 $\times \;10^{-1}$	**9.80** $\times \;10^{-1}$	1.25 $\times \;10^{-1}$	**9.80** $\times \;10^{-1}$	1.25 $\times \;10^{-1}$
**avgMAR ⇑**	3.26 $\times \;10^{-1}$	**9.60** $\times \;10^{-1}$	2.39 $\times \;10^{-1}$	-	-	-	-	-	-	-	-
**Density ⇑**	1.89 $\times \;10^{-1}$	**9.60** $\times \;10^{-1}$	1.85 $\times \;10^{-1}$	1.49 $\times \;10^{-2}$	1.49 $\times \;10^{-2}$	**9.60** $\times \;10^{-1}$	1.49 $\times \;10^{-2}$	**9.60** $\times \;10^{-1}$	1.59 $\times \;10^{-2}$	**9.60** $\times \;10^{-1}$	1.59 $\times \;10^{-2}$
**Centralization ⇑**	6.47 $\times \;10^{-2}$	9.32 $\times \;10^{-3}$	**1.18** $\times \;10^{-1}$	1.12 $\times \;10^{-1}$	1.12 $\times \;10^{-1}$	2.04 $\times \;10^{-2}$	1.12 $\times \;10^{-1}$	2.04 $\times \;10^{-2}$	1.11 $\times \;10^{-1}$	2.04 $\times \;10^{-2}$	1.11 $\times \;10^{-1}$
**Rand index ⇑**	**5.24** $\times \;10^{-1}$	-	-	-	-	-	-	-	-	-	-
**Adjusted Rand index ⇑**	**4.55** $\times \;10^{-1}$	-	-	-	-	-	-	-	-	-	-

⇑ signifies that a higher value of the corresponding validity index is better in determining the gene modules, while ⇓ denotes the reverse of the above statement. For each validity index, an entry denoted with bold font indicates that the corresponding method be the best performer in terms of the corresponding index (row-wise).

**Table 8:** Comparison of the proposed rule-based gene-module detection method and other existing geneset-based gene-module detection methods in the second simulation study for the cervical carcinogenesis dataset.

Validty Index	Proposed	wTOM[pcc]	wTOM[sc]	GTOM0[pcc]	GTOM0[sc]	GTOM1[pcc]	GTOM1[sc]	GTOM2[pcc]	GTOM2[sc]	GTOM3[pcc]	GTOM3[sc]
**avgDI ⇑**	**4.27** $\times \;10^{-1}$	Inf	Inf	Inf	Inf	Inf	-	Inf	-	Inf	-
**avgSW ⇑**	9.88 $\times \;10^{-2}$	3.67 $\times \;10^{-1}$	2.78 $\times \;10^{-1}$	9.15 $\times \;10^{-1}$	2.19 $\times \;10^{-1}$	**9.80** $\times \;10^{-1}$	-	**9.80** $\times \;10^{-1}$	-	**9.80** $\times \;10^{-1}$	-
**avgSC ⇓**	7.37 $\times \;10^{-1}$	9.92 $\times \;10^{-1}$	7.62 $\times \;10^{-1}$	9.80 $\times \;10^{-1}$	9.80 $\times \;10^{-1}$	9.80 $\times \;10^{-1}$	**1.27** $\times \;10^{-1}$	9.80 $\times \;10^{-1}$	1.47 $\times \;10^{-1}$	9.80 $\times \;10^{-1}$	1.48 $\times \;10^{-1}$
**avgCC ⇑**	2.58 $\times \;10^{-1}$	9.71 $\times \;10^{-1}$	2.43 $\times \;10^{-1}$	**9.80** $\times \;10^{-1}$	**9.80** $\times \;10^{-1}$	**9.80** $\times \;10^{-1}$	1.24 $\times \;10^{-1}$	**9.80** $\times \;10^{-1}$	1.36 $\times \;10^{-1}$	**9.80** $\times \;10^{-1}$	1.39 $\times \;10^{-1}$
**avgMAR ⇑**	3.26 $\times \;10^{-1}$	**9.71** $\times \;10^{-1}$	3.17 $\times \;10^{-1}$	-	-	-	-	-	-	-	-
**Density ⇑**	1.88 $\times \;10^{-1}$	**9.71** $\times \;10^{-1}$	2.77 $\times \;10^{-1}$	9.60 $\times \;10^{-1}$	9.60 $\times \;10^{-1}$	9.60 $\times \;10^{-1}$	1.64 $\times \;10^{-2}$	9.60 $\times \;10^{-1}$	2.00 $\times \;10^{-2}$	9.60 $\times \;10^{-1}$	2.04 $\times \;10^{-2}$
**Centralization ⇑**	6.72 $\times \;10^{-2}$	7.9 $\times \;10^{-3}$	**1.23** $\times \;10^{-1}$	2.04 $\times \;10^{-2}$	2.04 $\times \;10^{-2}$	2.04 $\times \;10^{-2}$	1.17 $\times \;10^{-1}$	2.04 $\times \;10^{-2}$	1.21 $\times \;10^{-1}$	2.04 $\times \;10^{-2}$	1.22 $\times \;10^{-1}$
**Rand index ⇑**	**5.54** $\times \;10^{-1}$	-	-	-	-	-	-	-	-	-	-
**Adjusted Rand index ⇑**	**3.67** $\times \;10^{-1}$					-	-	-	-	-	-

⇑ signifies that a higher value of the corresponding validity index is better in determining the gene modules, while ⇓ denotes the reverse of the above statement. For each validity index, an entry denoted with bold font indicates that the corresponding method is the best performer in terms of the corresponding index (row-wise).

**Table 9:** Summary of comparative performance between our proposed rule-module discovery method (in rows) over the traditional gene-module discovery methods using several existing similarity measures (in columns) for the original and simulated LUSC dataset (denoted as “LUSC”, “LUSC sm1”, and “LUSC sm2”, respectively) as well as the original and simulated cervical datasets (referred to as “Cervical”, “Cervical sm1”, and “Cervical sm2”, respectively).

Dataset	Method	wTOM[pcc]	wTOM[sc]	GTOM0[pcc]	GTOM0[sc]	GTOM1[pcc]	GTOM1[sc]	GTOM2[pcc]	GTOM2[sc]	GTOM3[pcc]	GTOM3[sc]
LUSC	**Proposed**	5-1-3	7-1-1	6-1-2	7-1-1	7-1-1	7-1-1	6-1-2	7-1-1	6-1-2	7-1-1
Cervical	**Proposed**	4-1-4	5-1-3	4-1-4	6-1-2	5-1-3	7-1-1	5-1-3	7-1-1	5-1-3	7-1-1
LUSC sm1	**Proposed**	5-0-4	7-0-2	6-0-3	7-0-2	5-0-4	5-0-4	5-0-4	6-0-3	6-0-3	5-0-4
LUSC sm2	**Proposed**	6-0-3	8-0-1	7-0-2	8-0-1	7-0-2	8-0-1	7-0-2	8-0-1	7-0-2	8-0-1
Cervical sm1	**Proposed**	5-0-4	5-0-4	6-0-3	6-0-3	6-0-3	8-0-1	6-0-3	7-0-2	6-0-3	7-0-2
Cervical sm2	**Proposed**	5-0-4	6-0-3	6-0-3	6-0-3	6-0-3	7-0-2	6-0-3	7-0-2	6-0-3	7-0-2

The entry at row X under column Y represents the win-draw-loss of X compared to Y.

## Supplementary Materials

The following are available online at https://www.dropbox.com/sh/uubqg9eq1qpeit3/AADmKhW08s8y7IzEf7joZWHZa?dl=0, Figure S1: Dendrogram of gene-modules through dynamic tree cut method by wTOM[sc] for the LUSC expression dataset; Figure S2: Plot of Silhouette width of gene-modules through dynamic tree cut method by wTOM[sc] for the LUSC expression dataset; Figure S3: Dendrogram of gene-modules through dynamic tree cut method by GTOM0[pcc] for the LUSC expression dataset; Figure S4: Plot of Silhouette width of gene-modules through dynamic tree cut method by GTOM0[pcc] for the LUSC expression dataset; Figure S5: Dendrogram of gene-modules through dynamic tree cut method by GTOM0[sc] for the LUSC expression dataset; Figure S6: Plot of Silhouette width of gene-modules through dynamic tree cut method by GTOM0[sc] for the LUSC expression dataset; Figure S7: Dendrogram of gene-modules through dynamic tree cut method by GTOM2[pcc] for the LUSC expression dataset; Figure S8: Dendrogram of gene-modules through dynamic tree cut method by GTOM2[sc] for the LUSC expression dataset; Figure S9: Dendrogram of gene-modules through dynamic tree cut method by the proposed method ConGEM for the LUSC expression dataset; Figure S10: Plot of Silhouette width of gene-modules through dynamic tree cut method by the proposed method ConGEM for the LUSC expression dataset; Figure S11: Dendrogram of gene-modules through dynamic tree cut method by wTOM[pcc] for the LUSC expression dataset; Figure S12: Plot of Silhouette width of gene-modules through dynamic tree cut method by wTOM[pcc] for the LUSC expression dataset; Text S13: Details about Statistical test for identifying differentially expressed genes; Figure S14: Dendrogram of gene-modules through dynamic tree cut method by the proposed method ConGEM for the Uterine cervical carcinogenesis methylation dataset; Figure S15: Several examples of calculating the proposed weighted measures; Text S16: The details of the used rule mining algorithm.
